# Evaluation of a Short-Term Digital Group Intervention to Relieve Mental Distress and Promote Well-Being Among Community-Dwelling Older Individuals During the COVID-19 Outbreak: A Study Protocol

**DOI:** 10.3389/fpubh.2021.577079

**Published:** 2021-04-09

**Authors:** Stav Shapira, Daphna Yeshua-Katz, Ganit Goren, Limor Aharonson-Daniel, A. Mark Clarfield, Orly Sarid

**Affiliations:** ^1^School of Public Health, Faculty of Health Sciences, Ben Gurion University of the Negev, Beer Sheva, Israel; ^2^PREPARED Center for Emergency Response Research, Ben Gurion University of the Negev, Beer Sheva, Israel; ^3^Department of Communication Studies, Faculty of Humanities and Social Sciences, Ben Gurion University of the Negev, Beer Sheva, Israel; ^4^The Spitzer Department of Social Work, Faculty of Humanities and Social Sciences, Ben Gurion University of the Negev, Beer Sheva, Israel; ^5^Faculty of Health Sciences, Medical School for International Health, Ben-Gurion University of the Negev, Beer Sheva, Israel; ^6^The Department of Geriatrics, McGill University, Montreal, QC, Canada

**Keywords:** older individuals, COVID-19, mental health and well-being, digital group interventions, cognitive-behavioral skills

## Abstract

Older individuals are at an increased risk of experiencing adverse social and health consequences due to both the COVID-19 pandemic and the measures taken to manage it, such as social distancing. To promote community-dwelling older individuals' well-being during this time, the aims of the current project are to develop effective strategies in order (a) to increase older individuals' digital literacy, and (b) to help them acquire behavioral and cognitive skills that will improve their coping abilities with the stressful situation created as a result of the pandemic, as well as reducing adverse mental health effects. The project comprises an intervention arm that includes digital group sessions for older individuals meant to improve their digital literacy, promote their effective coping, and relieve their mental distress and loneliness. Subjects receive a short-term (seven sessions), twice-weekly, digitally guided group intervention through Zoom (a video conferencing app), and WhatsApp (instant messaging app). The wait list control-group participants receive twice-weekly telephone calls from a research assistant during a parallel period. Web-based questionnaires are filled in pre- and post-participation. The effectiveness of the intervention will be analyzed by comparing pre- and post-measures, between intervention and control groups. This protocol offers a model for helping to support vulnerable populations during the COVID-19 pandemic. However, it is applicable regardless of the outbreak of a global health crisis or the imposition of lockdown rules; in fact, it has the potential to contribute to the social inclusion of vulnerable populations during routine times as well as during emergencies. Furthermore, ideas for future expansion include the integration of multilingual facilitators in order to reach seniors from underserved minority groups in various social contexts, even across borders.

## Introduction

The outbreak of the COVID-19 pandemic, which erupted in Wuhan, China, and which has spread quickly throughout the world, infecting millions, and claiming the lives of more than 2.5 million people (to date) (1), is at the center of concern for public health and healthcare agencies worldwide. Epidemiological investigations clearly indicate that increased mortality from COVID-19 is generally associated with older age and with the presence of chronic illnesses among this population, compromising their ability to deal with the myriad effects of the novel coronavirus (2, 3). Thus, the older population is especially at risk, in particular those who are frail and very old. Many countries around the world are advocating preventive measures such as social distancing, and governments are instructing at-risk populations, such as, older individuals, to self-isolate as much as possible by staying home. Although, these measures may be effective in preventing infection, morbidity, and death, they can also lead to increased loneliness and alienation (4, 5), potentially bearing dire health consequences both from a mental and physical health point of view (6). In many municipal jurisdictions, volunteers take care of delivering groceries and medications to the doorsteps of these populations; however, such efforts do not alleviate loneliness. An extensive body of knowledge suggests that loneliness among older people is strongly and independently associated with depression, high blood pressure, sleep disorders, prolonged and heightened stress responses, and even deterioration in cognitive function (6, 7). Other evidence suggests that loneliness among older adults is a major public health issue, and is associated with an increase in ongoing healthcare utilization (e.g., physician visits) (8) for chronic or acute non-COVID-19 conditions.

The use of new information and communication technology (ICT) (e.g., Skype, Facebook, WhatsApp, Zoom, and many more) offers a convenient alternative. In times such as the one we are currently living through, ICT enables the remote maintenance of social connections (9–12), and the conveyance of digital interventions over which therapeutic techniques and skills can be learned and practiced to promote effective coping, as well as alleviate loneliness, distress, and other mental health conditions (13–15). An extensive body of knowledge indicates the beneficial effects of digital interventions for diverse populations and conditions, using various communication modes. Therapeutic interventions delivered *via* digital platforms have been found to meet the same professional practice standards and outcomes as face-to-face delivery, and online delivery is particularly useful for people who are socially isolated (16–18). A meta-analysis that evaluated the clinical significance of guided digital interventions for patients with depression concluded that participating patients reported higher rates of remission compared to controls. In addition, older participants were more likely to respond to treatment than were younger participants (19). The results partially corresponded to those of another meta-analysis that examined the effect of digital interventions on loneliness and depression among older adults and reported a significant improvement only for the loneliness measure (20). Another study conducted in the U.S. described a significant improvement in measures of depression, quality of life, social support, and self-efficacy among older adults with diabetes who received a digital intervention guided by a nurse (21). However, the studies described above refer mostly to one-on-one rather than group interventions. Additionally, they were not conducted during a pandemic: a unique public health emergency that has necessitated social isolation and, as such, has posed an additional threat to the well-being and quality of life of at-risk populations such as older adults. Another recent study that was conducted in Germany among older individuals who had been traumatized as children found that a therapist-guided digital group intervention during which participants received cognitive-behavioral therapy (CBT) was associated with a substantial reduction in posttraumatic stress symptoms and with an increase in coping resources (22). Another study, conducted in South Africa, reported a significant reduction in loneliness levels among older adults who received a cognitive-behavioral group intervention *via* an instant messaging application (WhatsApp) (23).

There has been some debate regarding the effectiveness of group interventions compared with one-on-one activities among older adults (24), as well as regarding which modes of communication work better for delivering interventions to this population (25). Previous studies have usually referred to the utilization of a single digital platform such as email (26), forums and blogs (27, 28), and more recently, instant messaging (23) and videoconferencing (29). Given that each of these platforms has different, and often complementary functions, the integration of more than one platform for conveying therapeutic interventions also merits exploration. In the current protocol we evaluate the unique and common contribution of two digital platforms, WhatsApp (instant messaging app) and Zoom (video-conferencing app).

The importance of using ICT currently, during the COVID-19 crisis, can hardly be overstated; yet older people are likely to have what is commonly called an *age divide* (30), reflected in at least two levels of a digital divide between younger and older internet users. The first is internet connectivity, which differentiates between users and non-users, whereas the second is related to the skills and abilities required for ICT use (12, 31, 32). Moreover, a recent study documented a first-level digital divide within the senior citizen sector itself, the so-called *gray divide*, as reflected in connectivity and frequency of ICT use. That is, this study indicated that the major gap did not lie between what might be called the “pre-seniors” (50–59 years) and those younger, but rather between the “old seniors” (70+) and the rest of the populace (33). A study that explored the acceptance of health information technology among community-dwelling elders reported a variety of barriers related to familiarity and access, need for assistance, trust, privacy issues, design issues, and physical issues such as sight and hearing loss, loss of tactile senses, and cognitive and memory issues. The authors concluded that custom design and provision of relevant training can assist in overcoming these barriers (34). Nevertheless, the transition of many services to ICT may deepen the digital divide and increase social isolation if not properly tailored to the population of older adults (34–37). As such, future endeavors in the field of online therapeutic interventions should address this issue extensively if targeting older adults (34).

Despite the existence of the abovementioned substantial barriers, conducting interventions in an online format for older individuals appears feasible. Recent evidence shows growing rates of their embracing internet use (34). The COVID-19 pandemic has further highlighted how necessary and often inevitable it is to deliver therapeutic interventions through digital platforms. These platforms enable access to various health services that may otherwise be inaccessible (38), and if performed in a group manner, such interventions can reduce social isolation (39) at times such as these.

The aims of the current project are therefore to develop for at-risk populations, such as, older individuals, effective strategies in order to (a) increase their digital literacy, and (b) provide them with the tools and skills necessary for improving their coping abilities with the stressful situation created as a result of the pandemic, as well as reducing adverse mental health effects.

## Methods/Design

### Study Design and Setting

A prospective cohort intervention study among community-dwelling older adults.

### Inclusion and Exclusion Criteria

Eligible participants are adults aged 65 and older residing in the community who are proficient in Hebrew and can provide informed consent. Additional inclusion criteria are having an active internet connection, possessing at least one device that enables online communication, and having a minimal ability to operate this device (i.e., turning it on and off). Excluded from the study are participants who screen positive for major clinical depression on the Patient Health Questionnaire-9 (the PHQ9), defined as a sum score of 15< or score ≠ 0 on item 9. Participants are personally contacted by a member of the research team with clinical training in mental health care, in order to provide initial psychological support and information regarding appropriate further treatment options in the community. This procedure is a precautionary measure aimed at the early detection of, among other things, suicidal tendencies.

### Recruitment Procedure

Recruitment of study participants commenced in the month of April (2020) and will continue for 14 months, until June 2021, given the expectation that there will be additional COVID-19 waves (40). The main recruitment routes are advertisements to WhatsApp groups established by a local NGO responsible for promoting digital literacy among older adults. The advertisement contains information about the project and a link to a registration form for those interested in participating. In addition, we contacted relevant agencies responsible for the social care of older adults in several municipalities in the southern region of Israel (as these agencies have previous working collaborations with the research team) in an effort to widen the recruitment net. Social workers in these agencies were asked to refer relevant applicants, subject to the applicant's approval. In this manner, we have also been able to recruit participants with potentially low levels of digital literacy. All applicants are randomized on an 80:20 ratio into either the intervention or the control group, which is actually a waitlist for the intervention group, using a table of random numbers with no further constraints. The applicants referred by social services and those signing up voluntarily are randomized separately to ensure that an equal proportion of participants attained *via* both recruitment channels end up in both groups.

After randomization, research assistants contact the participants to: (1) fill in the questionnaires (T0), and (2) ensure that the ZOOM platform is installed on their computer or smartphone, and that their skill level of operating it is satisfactory. If necessary, remote assistance (*via* telephone) is provided in downloading and installing the app, and individual training is conducted in order for the participant to be able to independently hold a conversation. At this point, participants classified to the intervention group are assigned to small groups of up to seven participants. This sub-classification is done in accordance with three guidelines: (a) gender ratio; (b) maximum age difference of ~10 years; and (c) marital status balance (i.e., 50:50 ratio between participants who live alone vs. those who are married or have a common-law partner). The rationale underlying this sub-classification is an attempt to create small groups with a certain degree of basic similarity that will facilitate the participants' communication and connection to one another. We expect to see a higher application rate for women as their proportion among this age group (65<) is higher compared with men (41). Following this classification, the group moderator (a clinical social worker) is provided with his/her participants' contact details and information (name, phone number, age, and marital status). Each moderator establishes a WhatsApp group for the participants in his/her group in order to allow for ongoing communication between the participants themselves and/or with the moderators during and also between sessions.

### Digital Intervention Group to Relieve Mental Distress and Promote Well-Being

The online project comprises an intervention arm and wait list (control) group.

**Figure 1:**
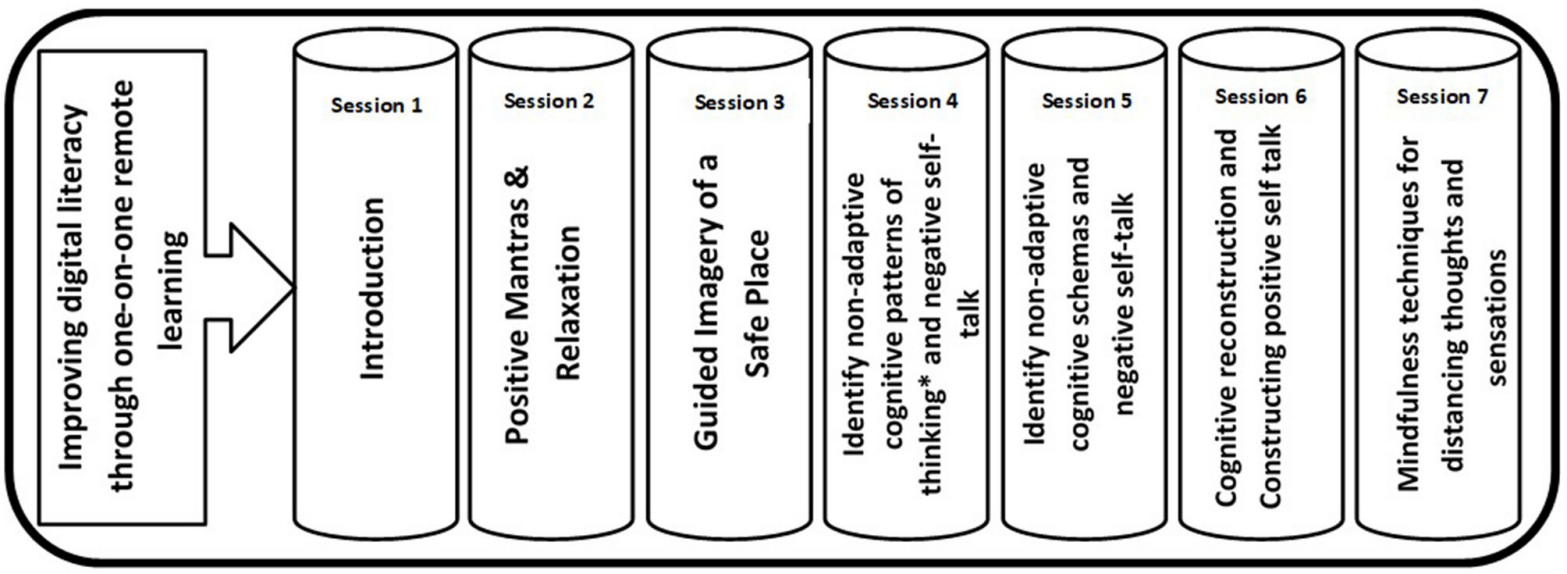
Skills and techniques addressed by the digital group intervention. Sessions are held *via* Zoom. Whatsapp is used to send participants supplementary materials such as voice recordings of relaxation, meditation, and guided imagery of a safe place to assist in self-practice. *Based on the work of Albert Ellis (43).

The intervention includes seven twice-weekly online guided group sessions *via* Zoom. In parallel, the participants can communicate with each other and with the moderator through a designated WhatsApp group. The main purpose of the intervention is to create a safe virtual learning space for participants to share their hardships in a supportive atmosphere, and acquire skills related to coping with the pandemic and the measures taken against it (i.e., social distancing). During each session participants learn and practice behavioral and cognitive techniques to reduce mental distress and promote well-being. The duration of each online session is between 60 and 90 min, and it consists of two parts: (a) a guided group discussion, and (b) learning and practicing cognitive-behavioral techniques and skills (CB intervention) such as, relaxation, guided imagery of a “safe place,” identifying non-adaptive cognitive schemas using Beck (42) and Ellis (43) categorization, cognitive restructuring, and constructing positive self-talk (15, 44, 45). Mindfulness techniques are taught as well, as part of positive self-talk and distancing strategies. All techniques are learned and practiced during the session (synchronized learning) under the guidance and supervision of the group's moderator, who also provides feedback to the participants. A schematic description of the entire intervention structure and the content, techniques, and skills delivered in each session is depicted in Figure 1.

The WhatsApp group is established 1 day prior to the first session. In addition to this platform's role as a channel for ongoing communication between the participants and their moderator, it also serves as (1) a tool for group management and data collection, by sending reminders and invitation links for the sessions, (2) a way to forward the links for the online questionnaires, and (3) a learning aid, *via* the sending of supplementary materials (video and audio files) for practicing the techniques taught in the sessions and tips for using Zoom and WhatsApp. During the first session, the moderator and the participants formulate a contract that defines the rules of conduct in the WhatsApp group in terms of content that can or cannot be shared, times of operation, and adherence to respectful discourse.

The online group moderators are clinical social workers with previous experience in delivering online cognitive-behavioral-based therapeutic interventions. A social work student in his/her final year is assigned to each moderator. The student takes part in all sessions and is responsible for assisting in administrative issues such as collecting data regarding emotional distress at the beginning and at the end of each session. Each moderator is provided with instruction once a week by a senior clinical social worker. The instruction sessions are focused on adapting the techniques and strategies to the needs of each group member.

The wait list control-group participants receive twice-weekly telephone calls (that is, “old-fashioned,” one-on-one, telephone calls, with no video accompaniment) from a research assistant during a parallel period.

**Figure 2:**
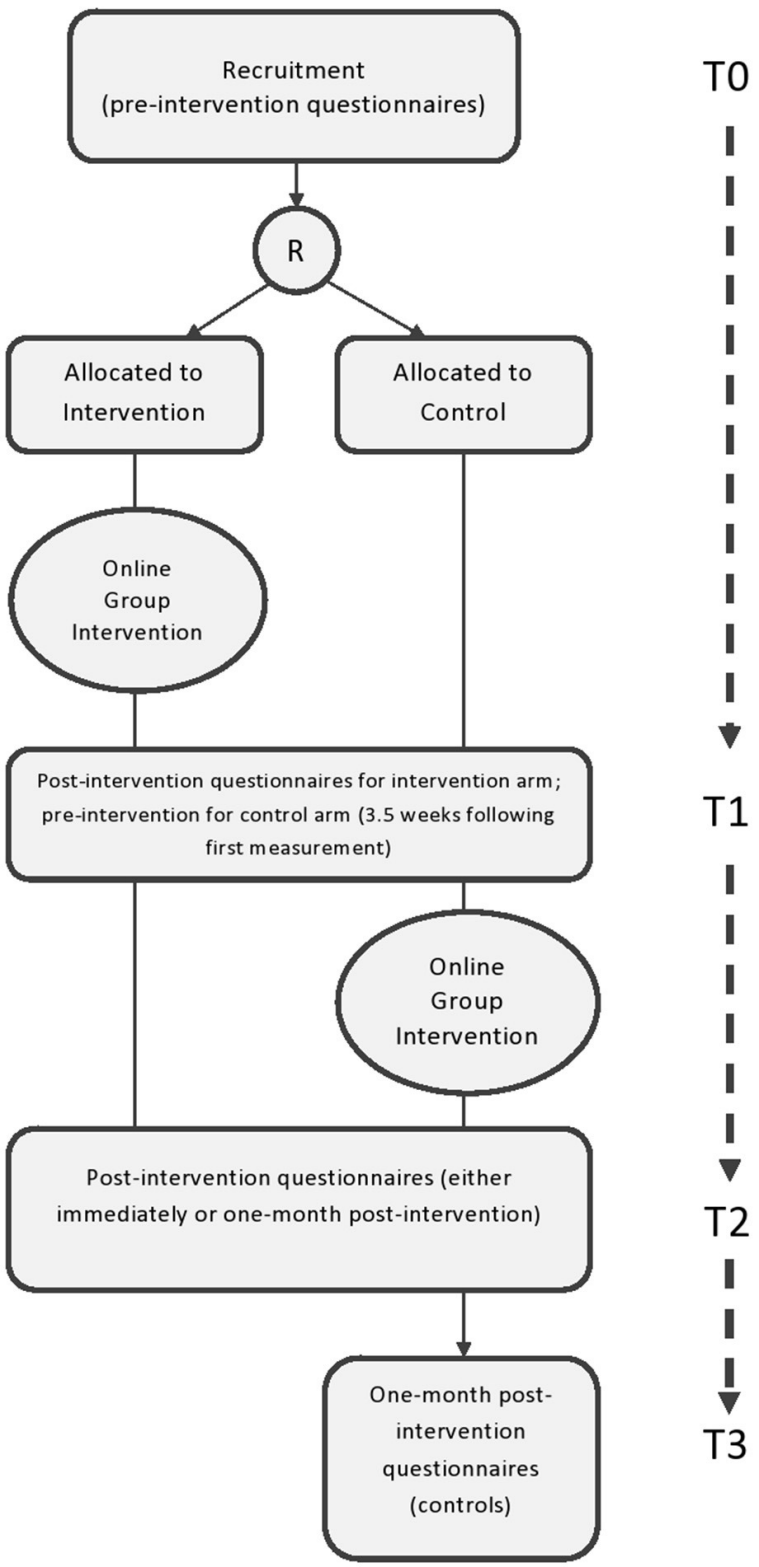
Participant flow chart and timeline.

All the participants fill out online questionnaires at three timepoints: pre-participation (T0); immediately post-participation (T1); and one-month post-participation (T2), in order to enable the evaluation of the intervention's effectiveness and the duration of impact. The link to the online questionnaire (web-based survey, https://www.qualtrics.com) is distributed by the groups' moderators to the participants' mobile phone or email, depending on their preference, near the start and end of the intervention: that is, no more than 48 h pre- or post-participation. The questionnaires assess digital literacy, social presence through technology, satisfaction with the use of computer-mediated communication, loneliness, self-reported depression symptoms, social support, resilience, self-efficacy, compliance with regular medication regime, adherence to social distancing practices, self-assessment of health status, and quality of life. Levels and changes of mental distress are monitored before and after each session. The waitlist control group participants are assessed twice before entering the intervention group, completing a total of four measurements (compared with three measurements in the intervention group) (see Figure 2).

### Measures to Assess the Effectiveness of the Online Intervention

Outcomes used to assess the effectiveness of the intervention procedure are described below. All measures are taken through web-based questionnaires at each of the abovementioned timepoints.

### Primary Outcome Measures

#### Mental Distress

Mental distress is monitored and assessed using two scales: (1) The Subjective Units of Distress Scale (SUDS) (46), to assess changes in distress levels pre- and post- each session. The SUDS provides a quick and simple way to measure distress in a given moment. The respondents are required to estimate the severity of their emotional distress before and after each session by giving a numerical value ranging from 0 (*totally relaxed*) to 10 (*highest distress that you ever felt*). The SUDS is a common tool for measuring the effect (pre- and post-) of therapeutic interventions (47, 48) and has been previously used among older individuals (49). (2) The short form of the Perceived Stress Scale (PSS-4) (50, 51). This scale is a commonly used tool which measures mental distress. The PSS-4 consists of four items that evaluate the degree to which individuals believe their life has been unpredictable, uncontrollable, and overloaded during the previous month, on a scale ranging from 0 (*never*) to 4 (*very often*). The responses are summed, resulting in a maximum total score of 16. Higher scores are correlated with more distress.

#### Loneliness

One of the main concerns related to protective measures such as, social distancing during the COVID-19 outbreak, especially among older individuals, has been an increase in loneliness. Our hypothesis is that participation in group sessions using a videoconferencing app will alleviate levels of loneliness. We use the Short Scale for Measuring Loneliness (52) which comprises three items examining perceptions related to lack of companionship, social exclusion, and social isolation. The response categories are coded as 1 (*hardly ever*), 2 (*some of the time*), and 3 (*often*). The responses are summed, with higher scores indicating greater loneliness.

#### Digital Literacy

The ability to operate and communicate *via* ICT is measured using questions from Israel's Central Bureau of Statistics (CBS) survey of web and computer use (53). These questions assess elements related to: (a) a first-level digital divide: the possession of digital devices such as computers, smartphones, etc., and an active internet connection package (two items); (b) a second-level digital divide: level of use and competence in operating web applications such as, email, search engines, social networking sites and apps, and using ICT such as, videoconferencing apps and instant messaging apps (10 items rated on a 5-point Likert scale ranging from 1 [*not at all*] to 4 [*to a high extent*]), and (c) barriers for ICT use (two multiple choice questions).

### Secondary Outcome Measures

#### Health and Well-Being

Perceptions of personal health status and health-related quality of life are examined *via* three questions from Israel's Central Bureau of Statistics (CBS) survey of health indicators (54). These questions relate to three health-related perceptions: (a) self-reported health, rated on a 4-point Likert scale; (b) self-reported independence in daily functioning, rated on a 5-point Likert scale; and (c) self-reported chronic morbidity, rated by a dichotomous question (yes/no) related to regular medication use.

Well-being is assessed using two questions from the OECD better life initiative (55) related to: (a) self-reported perception of quality of life (QOL), rated on a 5-point Likert scale; and (b) life satisfaction, rated on a 0–10 scale.

#### Self-Efficacy

The short form of the General Self-Efficacy Scale (GSE-6) (56, 57) contains six items, ranked on a 4-point Likert scale. The total score is calculated by finding the sum of all the items. For the GSE, the total score ranges between 6 and 24, with a higher score indicating more self-efficacy. The GSE is correlated with positive emotions, optimism, and life satisfaction. Negative coefficients have been reported for depression, stress, health complaints, burnout, and anxiety.

#### Resilience

The Conjoint Community Resiliency Assessment Measure (CCRAM) (58, 59) is a tool suitable for use in both routine and emergency times. The brief instrument includes 10 items measured on a 5-point Likert scale. The items were shown in a previous study (59) to form constructs of five factors: trust in leadership, collective efficacy, preparedness, place attachment, and social trust. The CCRAM score is composed of the average score of the constructs of these factors, each being assigned equal weight.

#### Social Support

Group interventions have the potential to enhance social support as a result of the interaction between the participants. We assess this notion using the Duke-UNC Functional Social Support Questionnaire (60) which is a validated tool for measuring social support among various populations, among them older adults (61). The tool contains eight items, rated on a 5-point Likert scale, that address both emotional support and functional support. An average score is calculated: The higher the average score, the greater the perceived social support.

#### Depression

High rates of depressive symptoms are generally common among older adults both during routine times and especially during and following crises. Depression and severity of relevant symptoms are assessed using a 9-item depression severity measure. This measure is part of the Patient Health Questionnaire (PHQ-9), and it is used as a diagnostic instrument for common mental disorders (62). The PHQ-9 scores each of the DSM-V criteria as 0 (*not at all*) to 3 (*nearly every day*). The responses are summed, resulting in a maximum total score of 27. Scores of 5, 10, 15, and 20 represent mild, moderate, moderately severe, and severe depression, respectively. The last item of the PHQ-9 targets suicidal inclinations and is utilized as a screening measure for suicidality in primary care. The PHQ-9 has previously been tested among the Israeli population (63).

#### Adherence to Social Distancing Practices

To assess the ability of the online intervention to increase adherence to social distancing practices, the research team developed 10 specific questions which are based on Israel's Ministry of Health guidelines, and which are published and regularly updated on the ministry's website (64). The participants are asked to report how often they performed eight activities such as, leaving their homes for stocking up on groceries, replenishing medication supplies, and exercising; how often they used others (family members or paid help) for those same tasks in order not to leave their homes; and how often they physically met with family and friends. The items are rated on a 5-point Likert scale ranging from 0 (*not at all*) to 4 (*once a day or more*). The responses are summed, resulting in a maximum total score of 40, with a lower value indicating higher compliance. Another item relates to the level of adherence to guidelines for using personal protective equipment (e.g., facial masks) and is assessed by a multiple-choice question. The last item relates to the perception of social distancing effectiveness in protecting participants from getting infected with COVID-19. This item is rated on a 7-point Likert scale, ranging from 1 (*not at all*) to 7 (*highly agree*).

### Sample Size Calculation

The Perceived Stress Scale (PSS-4) score is used as the primary outcome for sample size calculation. Harrer et al. (65) found a score of 7.43 (SD = 2.93) for the intervention group and 9.49 (SD = 3.06) for the control group in their study. They reported a decrease of 33% in the PSS-4 score post-intervention. We expect we will find similar results for our online intervention program, namely to find a decrease of between 20 and 30% in the total PSS-4 score. The target was set slightly lower in this study, because of the unique pandemic circumstances and social distancing practices which were absent in previous studies. Based on a power of 0.8, and a 2-sided alpha of 0.05, a sample size of 77 + 21 older people is needed, or 98 in total. This number was increased to *n* = 125 to account for a dropout rate of 25% in the intervention group and a 40% dropout rate in the control group.

### Quantitative Data Analysis

The differences in the outcome measures between the intervention and control groups will be measured using statistical tests comparing the change from baseline in outcome to immediately post-intervention and one-month post-intervention (for example, a paired *T*-test). Our participants' (self-enrolled and invited) data will be analyzed separately in light of the fact that potential differences in the degree of motivation may lead to a selection bias in the outcomes.

Multivariable regression models will also be conducted, and interaction effects between the study's primary and secondary outcomes will be examined. The analyses will be performed using SPSS (version 26, SPSS Inc., Chicago, IL, USA), an alpha level of 0.05 being accepted as significant. Analyses will be controlled for covariates such as gender, age, marital status, education, and household composition.

### Qualitative Data Analysis

All sessions will be recorded and transcribed verbatim. WhatsApp group correspondence will be mined as well. Both of these data components will be analyzed by content analysis, using ATLAS.ti (ATLAS.ti Scientific Software Development, Berlin). At least two researchers will scrutinize the qualitative data and participate in their interpretation.

### Ethical Considerations

Using internet-based platforms to deliver mental health interventions raises a number of ethical issues and challenges. First, in order to ensure confidentiality and privacy during the online sessions and minimize cybersecurity risks, the collection and storage of data comply with the Israeli Protection of Privacy Regulations (Data Security): All data collected will be stored in a secured server and only the main investigators will have access to the final datasets. The second issue relates to distress management or the risk that the topics discussed during the sessions may exacerbate distress among some participants. This risk is addressed by guiding the group's moderators, who are trained clinicians, to monitor the participants' affect and if deem necessary to conduct a brief risk assessment and provide additional support following the end of the session.

## Discussion

This study aims were to develop a short-term online group intervention utilizing Zoom and WhatsApp to increase digital literacy, relieve adverse mental health effects and promote better coping and well-being among community-dwelling older individuals who have been shut in during the COVID-19 pandemic. Promoting the well-being of older people during a global health crisis requires an understanding of the target group and the specific hardships with which they struggle during routine and emergency situations. These individuals may find it difficult to navigate through the sea of online resources, and are thus highly vulnerable in scenarios where social distancing and isolation are required, and in which digital tools become a primary source of communication. Thus, developing an intervention that enhances both digital literacy and coping skills can be central to their resilience and well-being during such times, and provide valuable information regarding their abilities to adapt and strive in times of public health crises. Identifying those who are the better adaptors and those who struggle with difficulties can enhance preparedness for future events.

Furthermore, as older people are generally considered particularly susceptible to loneliness and mental distress (66, 67), the digital and coping skills acquired can be applied also during routine daily life or during future disease outbreaks. Thus, this project stands to make an even greater contribution in terms of the known and global phenomenon of social isolation among older individuals, and its potentially dire health and social consequences (66–68).

From a broader public health perspective, providing older individuals with the knowledge and ability to independently communicate online with their significant others and/or with various caretakers can contribute, even if indirectly, to their adherence to protective guidelines of physical distancing and to minimizing their exposure to the coronavirus or other infectious diseases.

If this protocol is proven effective, it can be expanded to other populations such as, older individuals living in assisted living frameworks or nursing homes, people with disabilities, or any population at high risk of experiencing mental distress during a continuous public health emergency (69, 70) such as the COVID-19 pandemic. Additionally, although the current protocol is addressed solely to Hebrew-speaking older individuals, another future potential expansion could involve the integration of multilingual facilitators in a specific social context in order to reach underserved older adults from other ethnic groups who might benefit from both improving digital literacy and coping skills. For example, in the Israeli context, the integration of Arabic, Russian, and Amharic speaking moderators would help make the intervention more accessible, and more socially sensitive (71) to all of the sub-populations that comprise Israeli society.

The surge of interest in and acceptance of digital tools among both health and social care providers and consumers that has been triggered by the COVID-19 global crisis (72) offers a unique and important opportunity to explore the effectiveness and full potential of various digital initiatives that offer support and mental health care (73). In this regard, developing high-quality protocols to evaluate these digital initiatives is particularly crucial in order to provide clear evidence of their efficacy and benefits on the one hand, and/or to expose potential risks and pitfalls on the other. We feel that our protocol offers a robust design for the evaluation of a digital group intervention to improve mental health indicators. It can easily be adapted to evaluate other digital initiatives in this field or similar fields (e.g., “lifestyle interventions,” such as those focused on physical exercise, diet, etc.,) (74) that might also be impacted during long periods of isolation.

### Challenges and Limitations

There are several challenges in the implementation of this intervention. First, the current recruitment procedure may potentially lead to a selection bias. Using digital platforms as a primary source for recruitment may lead to the exclusion of ICT non-users, people of low socioeconomic status, and other marginalized groups who may well be most in need of these kinds of interventions. It is our current goal to establish a structured infrastructure for participants' enrollment through Israel's Ministry of Social Affairs and Social Services, as well as through local municipalities. Doing so will allow for an internal mapping of older individuals across Israel in order to locate people who live alone, and/or have background morbidities (i.e., people who constitute the most vulnerable subgroup of older individuals in the current pandemic context). In the event of a second outbreak, this kind of mapping will enable a rapid implementation of the protocol.

Another challenge relates to the time and resources required to offer such interventions. Although, we believe that the benefits of the program detailed above outweigh these challenges, we see much importance in investing more research to reduce costs and make the program sustainable.

And finally, the scope of the intervention is also worth discussing. As this intervention focuses on mental health as a way to promote well-being, it may have a limited impact on individual lifestyle behaviors in the long run, especially if learned strategies are not practiced on a daily basis. Important other determinants of health and well-being such as physical exercise, good dietary management, and cultural activities – the likely lack of which have all had an impact during the pandemic outbreak (74, 75) – are not addressed by the current online intervention, although, there may be some indirect positive effect of our program on older people's approach to these issues. To this end, we did use the WhatsApp groups as a channel to inform participants of other initiatives and events in these domains. Hence, the digital group intervention offered here is only one element of an integrated, system-wide approach required for the successful support of vulnerable populations during an ongoing public health emergency such as that which has been caused by the current eruption of COVID-19.

## Ethics Statement

Ethical approval to conduct this study was obtained from the Ben Gurion University of the Negev (BGU) Human Subjects Research committee (ID: 1885-1). Informed consent will be obtained from all study participants at baseline. Research assistants will send the form *via* email or WhatsApp and the participants will then provide their written informed consent.

## Author Contributions

SS, DY-K, GG, LA-D, AMC, and OS are members of the research team and contributed to the design of the study. SS, DY-K, LA-D, AMC, and OS developed the study protocol. OS and GG developed the cognitive-behavioral intervention protocol. SS wrote the manuscript. All authors provided comments and approved the final manuscript.

## Conflict of Interest

The authors declare that the research was conducted in the absence of any commercial or financial relationships that could be construed as a potential conflict of interest.

## Abbreviations

COVID-19: Coronavirus disease 2019
ICT: information and communication technology
CBT: Cognitive behavioral training
CBS: Central Bureau of Statistics
OECD: Organization for Economic Co-operation and Development
QOL: Quality of life
DSM-V, Diagnostic and Statistical Manual of Mental Disorders: Fifth Edition.
